# Supplementary figures and images for: Genetic variation of putative myokine signaling is dominated by biological sex and sex hormones
Source: eLife. 2022 Apr 13;11:e76887. doi: 10.7554/eLife.76887 (PMC9094747; doi:10.7554/eLife.76887)

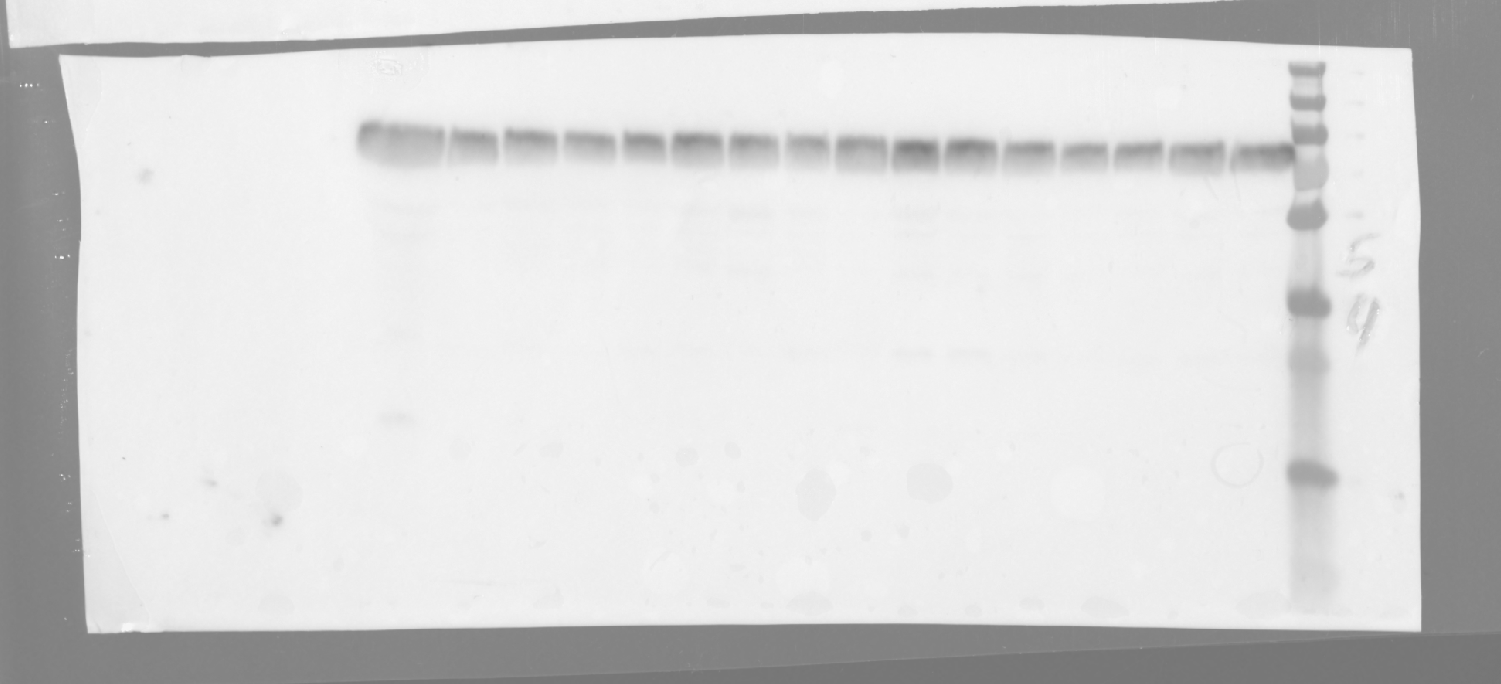

Supplement: Figure 1—source data 1. [file elife-76887-fig1-data1.zip › bActin raw gel.tif]

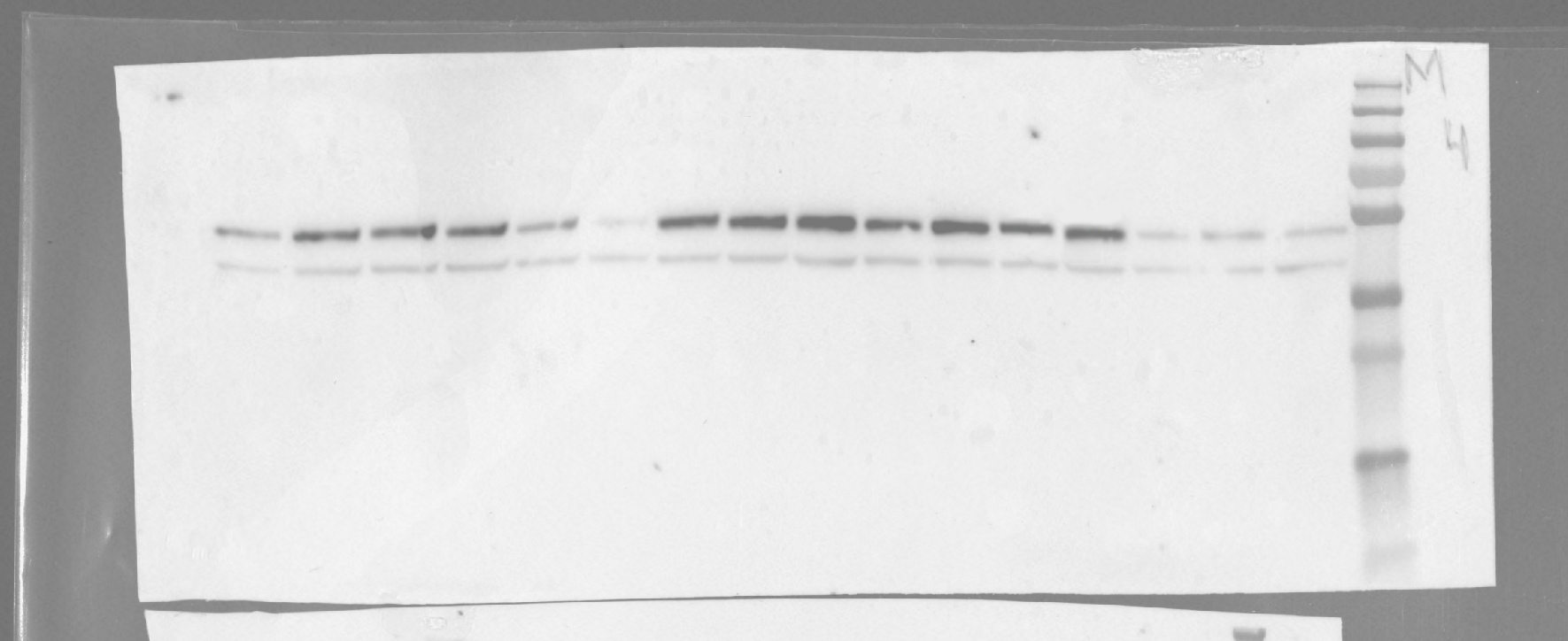

Supplement: Figure 1—source data 1. [file elife-76887-fig1-data1.zip › Myostatin RAW GEL.tif]
